# Diagnostic challenges of longstanding nasal cutaneous tuberculosis in an endemic setting: a case report

**DOI:** 10.11604/pamj.2025.51.95.48456

**Published:** 2025-08-18

**Authors:** Anke van der Linden, Eugenne Elliot, Rehuel Borstlap

**Affiliations:** 1 Department of Otorhinolaryngology, Head and Neck Surgery, Robert Mangaliso Sobukwe Hospital, Kimberley, Northern Cape, South Africa

**Keywords:** Granulomatous disease, tuberculosis, lupus vulgaris, nasal cutaneous tuberculosis, case report

## Abstract

Nasal cutaneous tuberculosis (TB) is a rare manifestation of extrapulmonary tuberculosis and presents a diagnostic challenge, particularly in its paucibacillary form. As demonstrated in this case, achieving laboratory-confirmed diagnosis in paucibacillary TB remains a significant challenge, often resulting in missed or delayed diagnoses and increased severity of disease on presentation. We report an atypical case involving a 19-year-old male with extensive nasal destruction progressing for fourteen years. In this case, lupus vulgaris was ultimately diagnosed after extending the tissue culture duration beyond 35 days, despite prior exclusion of TB as the cause. The patient completed antituberculosis therapy with resolution of the active disease, and he was referred for further management of his facial deformity. The delayed diagnosis led to significant tissue destruction, which could have been prevented with earlier confirmation and treatment of the infection. We propose that, in diagnostically difficult cases, the investigating team consider extending the specimen culture time to the maximum before definitively excluding tuberculous disease.

## Introduction

Cutaneous tuberculosis (CTB) is diagnostically elusive due to the condition's diverse clinical morphology, uncommon presentation, and the frequent difficulty in obtaining microbiological confirmation [1,2]. CTB is rare, estimated to account for approximately 1-2% of all extrapulmonary tuberculosis cases [3]. Lupus vulgaris (LV) is a paucibacillary form of CTB, a classification that also includes tuberculosis verrucosa cutis and tuberculids. *Mycobacterium tuberculosis* is the predominant causative agent.

The gold standard for diagnosis of CTB is a successful tissue culture [3], yet obtaining laboratory confirmation or a positive culture in paucibacillary CTB remains challenging. This can result in delayed or missed diagnoses, disease progression, and increased severity of disease on presentation - as was seen in this case. Despite the prevalence of CTB, nasal LV is an uncommon presentation, with cases of absent external nasal structures secondary to LV being even more rare. We present one such uncommon case of lupus vulgaris affecting the external nose.

## Patient and observation

**Figure 1:**
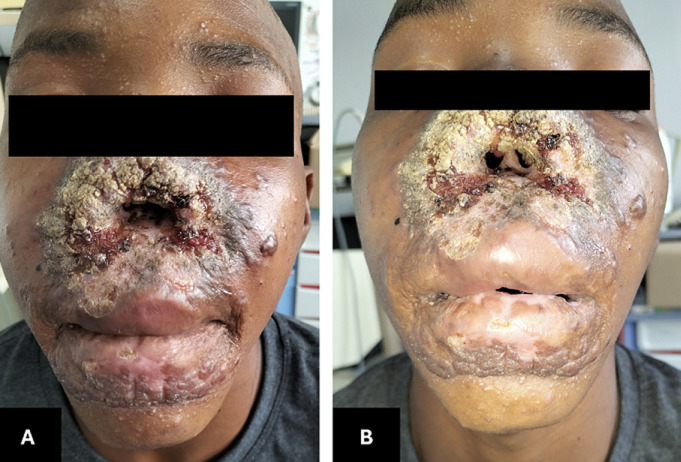
A, B) clinical appearance at the first visit: significant centrofacial mutilation and absence of anterior nasal structures, with ulcerating, vegetative reddish-brown plaques and extensive crusting

**Patient information:** an otherwise healthy 19-year-old male was referred from a primary care facility with absent external nasal structures and centrofacial granulomatous skin lesions progressing for fourteen years (Figure 1).

**Clinical findings:** he first presented to a primary care facility in 2014 (age ten), five years after he first developed lesions on his upper lip and philtrum. The disease had since progressed to involve his perioral region, inferior nasal skin, and his anterior nasal septum, without septal perforation.

**Timeline of the current episode:** upon presentation in 2014, his first workup was initiated. Extensive investigations, including tissue biopsies for histology and TB cultures, were conducted. Prior to establishing a diagnosis the patient defaulted follow-up. Over the next decade (2014-2023), the patient underwent several diagnostic evaluations but repeatedly defaulted before a definitive diagnosis could be made. Throughout this time, he had numerous investigations performed, some of which included new tissue biopsies for histology and TB cultures (2014, 2018, and 2023) (Table 1). Beyond TB, other causes investigated included sarcoidosis, leprosy, and atypical fungi [4].

**Table 1:** summary of investigations and results

Investigations	2014 - Primary Presentation	2015 - Second Presentation	2018 - Third Presentation	2023 - Latest Presentation
**Inflammatory Markers (WCC, ESR, CRP)**	WCC, CRP Normal ESR 45 (Normal 0-10)	WCC Normal ESR 36	WCC, CRP Normal ESR 41	WCC, CRP, and ESR Normal (0-10)
**HIV and Syphilis Serology**	Negative	Negative	Negative	Negative
**Liver and Renal Functions**	Normal	-	Normal	Normal
**ACE**	-	-	-	Slightly raised; ACE 73 (Normal 8-52)
**Autoimmune Studies**	-	-	C3, C4, ANAIF, ADNAIF, ANAEL all negative	ANAIF, AP3EL, AMPEL all negative
**CXR**	Normal	-	Normal	Normal
**High Resolution CT**	Defaulted	-	Defaulted	Done after diagnosis of TB was made; no features of sarcoidosis
**Sputum GXP**	Negative	Negative	Negative	Negative
**Tissue GXP**	Negative	-	Negative	Negative
**Tissue TB Culture**	Negative growth result at 35 days; Negative Auramine O Stain	-	Negative at 35 days	**Biopsy 1 - November 2023:** Negative at 35 days; Positive at 42 days **Biopsy 2 - December 2023:** Negative at 35 days
**Histopathology**	-Hyperplastic stratified squamous epithelium -No granulomatous inflammation or malignant Stigmata -Findings have a wide differential diagnosis -Stains for fungal elements and acid-fast bacilli are negative	-	-Necrotising granulomatous inflammation with epithelial thickening and a dense dermal inflammatory process -Findings have a wide differential diagnosis -Stains for acid-fast bacilli and fungal elements are negative	**Biopsy 1 - November 2023:** -Hyperparakeratotic, hyperplastic stratified squamous epithelium, overlying stroma which shows extensive granulomatous inflammation -Granulomas are mostly made up of epithelioid cells, with no caseous necrosis -Findings have a wide differential diagnosis -Stains for acid-fast bacilli and fungal elements are negative **Biopsy 2 - December 2023:** -Skin with ulcerated epidermis; the ulcer is covered by necrotic debris, mixed with neutrophils and fibrin. Epidermis adjacent to ulceration shows hyperplastic changes -The dermis illustrates extensive involvement by granulomatous inflammation, comprising of Langhans and foreign body giant cells, lymphocytes and epithelioid cells -The dermis surrounding granulomatous inflammation shows a dense, lymphoplasmacytic inflammatory infiltrate -Features compatible with granulomatous inflammation with histological differential also including sarcoidosis, tuberculosis, leprosy as well as syphilis -No fungal elements or acid-fast bacilli identified

**Diagnostic assessment:** he re-presented in November 2023 and a new, comprehensive workup was done. Investigations included radiological imaging, blood tests, and new tissue biopsies of his perinasal region. Specimens were sent for histological analysis, TB culture, and TB nucleic acid amplification tests (TB NAAT) [5]. X-ray imaging and laboratory markers of infection and inflammation were non-specific, with normal results except for a moderately raised angiotensin-converting enzyme (ACE) level. The TB NAAT tests were negative again, and the histopathological analysis of the new specimens delivered similar results to those obtained in 2014 and 2018, with negative stains and no conclusive evidence of TB (Table 1). Considering all the previous negative culture findings, repeat biopsies were taken for histology and culture. While awaiting these results, our patient was booked for a high-resolution computed tomography (CT) scan of his chest to exclude sarcoidosis, which remained a possible diagnosis.

**Therapeutic interventions:** after collecting the last samples, a trial of standard antituberculosis therapy was initiated while awaiting the final culture and CT scan results, given the persistent clinical suspicion of TB [6].

**Figure 2:**
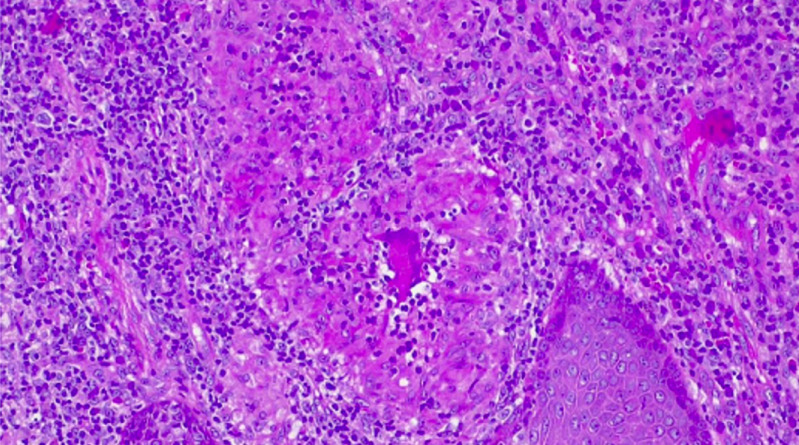
histologic image of the fourth submitted tissue specimen (December 2023): the first specimen with histopathological features more suggestive of cutaneous tuberculosis

**Figure 3:**
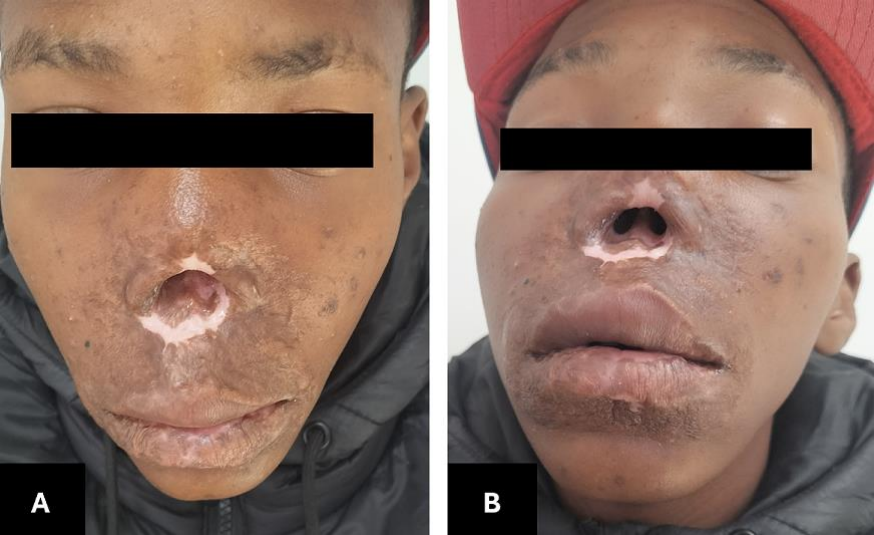
A, B) post-treatment images with healed skin lesions, residual nasal defect evident, but overall, markedly improved appearance

**Follow-up and outcome of interventions:** the TB culture, incubated using the Becton Dickinson mycobacteria growth indicator tube (BD MGIT), was extended and became positive on day 42. The second biopsy taken in December 2023, though negative for TB on culture at 35 days, was the first of four specimens that had histological features suggestive of TB (Figure 2, see Table 1). The patient completed TB treatment and showed remarkable recovery of his facial lesions in 2024 (Figure 3). After clinical resolution of his TB, he was referred for reconstructive surgery, also to our dermatology department for annual follow-up and screening for recurrence or carcinomatous changes.

**Patient perspective:** the patient was grateful that a diagnosis was finally made and that the treatment had such a good effect.

**Informed consent:** written informed consent was obtained from the patient for publication.

## Discussion

LV is a paucibacillary, post-primary form of CTB, occurring in individuals with a moderate to high degree of immunity. Hematogenous seeding is thought to be the main method of spread from the primary site, but it can also occur secondary to autoinoculation or lymphatic dissemination. Despite the prevalence of TB and its global burden of disease, a patient presenting with absent external nasal structures secondary to destruction caused by sinonasal LV is extremely rare. LV is characterized by its infrequency and clinical polymorphism, often mimicking other granulomatous or neoplastic processes, contributing to its diagnostic difficulty. The gold standard for diagnosis of CTB is a successful tissue culture and susceptibility testing to exclude multidrug-resistant and extensively drug-resistant tuberculosis (MDR/XDR TB).

Resource constraints have necessitated limiting the culture duration to 35 days [7], but it is not long enough to exclude all paucibacillary infections, as is demonstrated by this case. In similar situations, extending the incubation time should be considered and can be requested from the laboratory. The breakthrough in this case was achieved by prolonging the specimen's culture time. Despite adequate specimen size and extensive diagnostic efforts, all four TB cultures were negative at 35 days. Currently, BD MGIT machines can only detect bacteria at 10^1 to 10^2 bacilli/mL [8,9]. In the case described above, multiple histopathological, laboratory, and imaging tests were also nonspecific. This, combined with the negative TB culture result at 35 days, previously led clinicians to exclude TB as the potential cause. It was only when the TB culture duration was extended beyond the routine culture time that a positive culture result was finally obtained on day 42 from the specimen collected in November 2023 (Table 1).

## Conclusion

We recommend that in diagnostically complex cases, clinicians consider extending the specimen culture time to the maximum before definitively excluding TB. This is especially relevant in areas of high TB prevalence [10] where there is a high index of suspicion of CTB, combined with a negative culture result at 35 days and a lack of other conclusive diagnostic evidence. Positive culture results allow directed treatment; negative extended culture results will exclude TB as the diagnosis, for the treatment of alternative causes of the mutilating effect could accelerate multiplication of TB bacilli and exacerbate tissue damage. This is applicable to other forms of extrapulmonary TB; however, this consideration is especially relevant in challenging cases of cutaneous disease, as well as other rare or low-yield conditions that can be attributed to TB infection. After obtaining adequate samples, a trial of antituberculosis therapy can be considered awaiting pending results, as clinical improvement is often rapid and marked when TB is correctly identified and treated.
